# Supplementary material for: MiRNA-Regulated Pathways for Hypertrophic Cardiomyopathy: Network-Based Approach to Insight into Pathogenesis
Source: Genes (Basel). 2021 Dec 18;12(12):2016. doi: 10.3390/genes12122016 (PMC8701189; doi:10.3390/genes12122016)
Supplement: Supplementary file 1 [file genes-12-02016-s001.zip › Suppl_4.pdf]

Supplementary File 4: Test for the uniqueness of miRNA-Reactome bigraph characteristics

A graph $G = (V, E)$ consists of two sets V and E. The elements of V are the vertices and the elements of E are the edges of G.

Let M denotes a set of investigated miRNAs and Rp denotes a set of Reactome pathways, and given a function $f(x)$:

$$f(m_i \in M) = \{\text{key target genes}\}$$

$$f(r_i \in Rp) = \{\text{genes in pathway } r_i\}$$

then $B = (V, E)$ is bigraph, where:

$$V = M \cup Rp$$

$$E \subseteq \{(m_i, r_j) \mid m_i, r_j \in V \text{ and } f(m_i) \subset f(r_j)\}$$

The observed degree distribution $F(B)$ of vertices $v_i \in Rp$ is:

Degree (j)	1	2	3	4	5	6	7
Amount of nodes $(\lvert\{v_i \lvert deg(v_i) = j\}\rvert)$	216	49	36	18	10	6	3

We assume that if the Reactome pathways with high degree are unique for investigated miRNAs, then the observed degree distribution should be shifted to the right relative to the distribution for random selected miRNAs.

Thus, the H_0-hypothesis is $F(B) = F(B_{random})$, where B_{random} is bigraph $B_{random} = (V_r, E_r)$ and:

$$V_r = \hat{M} \cup Rp, \hat{M} \subset MT, |\hat{M}| = |M|$$

where MT is a set of miRNAs indexed in miRTarBase, and $\hat{M}$ is a set of random selected miRNAs from MT

$$E_r \subseteq \{(m_{r_i}, r_{r_j}) \mid m_{r_i}, r_{r_j} \in V \text{ and } f(m_{r_i}) \subset f(r_{r_j})\}$$

We can test the H_0-hypothesis using Chi-square statistics, with the Monte-Carlo's method for estimation of the expected frequencies of amount of nodes with $deg(v_{r_i}) = j, v_{r_i} \in V_r$.
For this:

1. we randomly choose with replacement 8 miRNAs from miRTarBase so that:
   (a) 95%CI for a number of selected miRNA targets $\subset$ 95%CI for a number of investigated miRNA targets
   (b) minimum $\mathrm{LCC_{miRNA_i}}$ size of selected miRNAs $\geq$ minimum $\mathrm{LCC_{miRNA_j}}$ size of investigated miRNAs
   (c) minimum number of key target genes of $\mathrm{miRNA_i}$ from selected miRNAs $\geq$ minimum number of key genes of $\mathrm{miRNA_j}$ from investigated miRNAs
2. construct a B_{random} and estimate the expected frequencies of amount of nodes with $deg(v_i) = j$ by mean value
3. repeat steps 1-2 10000 times

As a result, we got the following estimates of $F(B_{random})$:

Degree (j)	1	2	3	4	5	6	7
Amount of nodes ($\lvert\{v_{r_i} \lvert deg(v_{r_i}) = j\}\rvert$) (+/- 95%CI)	194.9 (179.8-205.4)	88.7 (82.5-99.7)	40.5 (35.4-45.3)	16.8 (14.5-18.9)	6.9 (5.7-8.7)	3.6 (2.8-4.9)	2.5 (1.7-3.8)

The p-value is:

$$P(x > \chi^2) = P(x > \sum_{j=1}^{j=5} \frac{(|\{v_i|deg(v_i) = j\}| - |\{v_{r_i}|deg(v_{r_i}) = j\}|)^2}{|\{v_{r_i}|deg(v_{r_i}) = j\}|}) = 5.9 * 10^{-4}$$
