# Supplementary material for: MiRNA-Regulated Pathways for Hypertrophic Cardiomyopathy: Network-Based Approach to Insight into Pathogenesis
Source: Genes (Basel). 2021 Dec 18;12(12):2016. doi: 10.3390/genes12122016 (PMC8701189; doi:10.3390/genes12122016)
Supplement: Supplementary file 1 [file genes-12-02016-s001.zip › Suppl_5.pdf]

Supplementary File 5: Estimation of the probability of obtaining the signaling pathways

1 The module pinpointed after the first iteration of the Greedy search

In the article, we show that 8 out of 12 miRNAs target three TGF-beta/SMAD signaling pathways:

$$TGF_{set} = \{(R{-}HSA{-}170834), (R{-}HSA{-}2173793), (R{-}HSA{-}2173796)\}$$

What is the probability that a pathway be connected with $1, 2, 3, \ldots, n$ miRNAs? Or in other words, what is the probability that a node heve degree k, where $k \in \{1, 2, .., n\}$? Each node has (n) tries to get edges. Each try is a success with probability p. The binomial distribution gives us the probability that a node has degree k:

$$Bin(n, k, p) = \binom{n}{k} * p^k * (1 - p)^{(n-k)}$$

where p for the $pathway_i$ could be above estimate, as:

$$p_i = \frac{(number\ of\ miRNAs\ targeted\ the\ pathway_i)}{(number\ of\ miRNAs)}$$

Straightforward, that for our purposes the sentence "the n miRNAs target set of pathways" is equal to "each of the miRNAs targets at least one pathway from the set". Thus, for the TGF_{set} we could rewrite the p calculation as:

$$p_i = \frac{(number\ of\ miRNAs\ targeted\ at\ least\ one\ pathway\ from\ TGF_{set})}{(number\ of\ miRNAs)} = 0.35$$

Now, defining the $p-value$ as the probability of getting k or more miRNAs targeting at least one pathway from TGF_{set}, we can calculate it as:

$$p-value = P(card\{miRNAs_{pathwai_i}\} \geq 8) \sim Bin(12, 8, 0.35) = 0.0062$$

where:

$card$ is the cardinality of a set

$\{miRNAs_{pathwai_i}\}$ is set of miRNAs targeted any $pathway_i \in TGF_{set}$

2 The module pinpointed after the second iteration of the Greedy search

The second module consists of two pathway clusters: $first$ – pathways linked to hsa-miR-1-3p and hsa-miR-497-5p *second* – pathways linked to hsa-miR-133a-3p and hsa-miR-199a-3p and two "*bottleneck*" pathways linked via hsa-miR-199a-3p to the first cluster and via hsa-miR-497-5p and hsa-miR-1-3p to the second cluster.

We repeated the above reasoning for each of these clusters:

$$p-value_{first} = P(card\{miRNAs_{pathwai_i}\} \geq 2) \sim Bin(12, 2, 0.39) = 0.91$$

$$p-value_{second} = P(card\{miRNAs_{pathwai_i}\} \geq 2) \sim Bin(12, 2, 0.17) = 0.35$$

$$p-value_{bottleneck} = P(card\{miRNAs_{pathwai_i}\} \geq 3) \sim Bin(12, 3, 0.23) = 0.11$$

And we calculated the p-value for the all module with Bonferroni correction for 3 tests as:

$$p-value_{module} = (p-value_{first} * p-value_{second} * p-value_{bottleneck}) * 3 = 0.11$$

3 Code available

https://github.com/GJOsmak/miRNET_HCM/Code/pathway_test.ipynb
