# Supplementary material for: MiRNA-Regulated Pathways for Hypertrophic Cardiomyopathy: Network-Based Approach to Insight into Pathogenesis
Source: Genes (Basel). 2021 Dec 18;12(12):2016. doi: 10.3390/genes12122016 (PMC8701189; doi:10.3390/genes12122016)
Supplement: Supplementary file 1 [file genes-12-02016-s001.zip › Suppl_6.pdf]

Supplementary File 6:
Preliminary run of Greedy search using a reduced list of miRNAs

We constructed the miRNA-pathway network for miRNAs hsa-miR-29a, hsa-miR-93, hsa-miR-133a, hsa-miR-199-3p, hsa-miR-221, hsa-miR-222, hsa-miR-451 and hsa-miR-497 (without taking into account their -3p/5p strand specificity unless explicitly stated) in order to identify those of the signaling pathways that are regulated by the largest number of these miRNAs. As a result, we obtained a connected network in which all miRNAs were present.

We used the Greedy algorithm to search for Reactome pathways connected to the majority of selected miRNAs (Figure. 1). At the first iteration of the algorithm, we pin-pointed two pathways: “Transcriptional activity of SMAD2/SMAD3:SMAD4 heterotri-mer” and “SMAD2/SMAD3:SMAD4 heterotrimer regulates transcription”, which were overrepresented by key target genes of hsa-miR-29a, hsa-miR-93, hsa-miR-222, hsa-miR-451 and hsa-miR-497. The first pathway includes the second one, therefore only the “Transcriptional activity of SMAD2/SMAD3:SMAD4 heterotrimer” pathway was further analyzed. To perform the second iteration of the Greedy search we excluded the above-mentioned SMAD2/SMAD3:SMAD4 dependent pathways and connected miR-NAs. As a result, we pinpointed the pathway “Signaling by VEGF”, which was overrepresented by targets of the rest of miRNAs hsa-miR-133a, hsa-miR-199a-3p and hsa-miR-221.

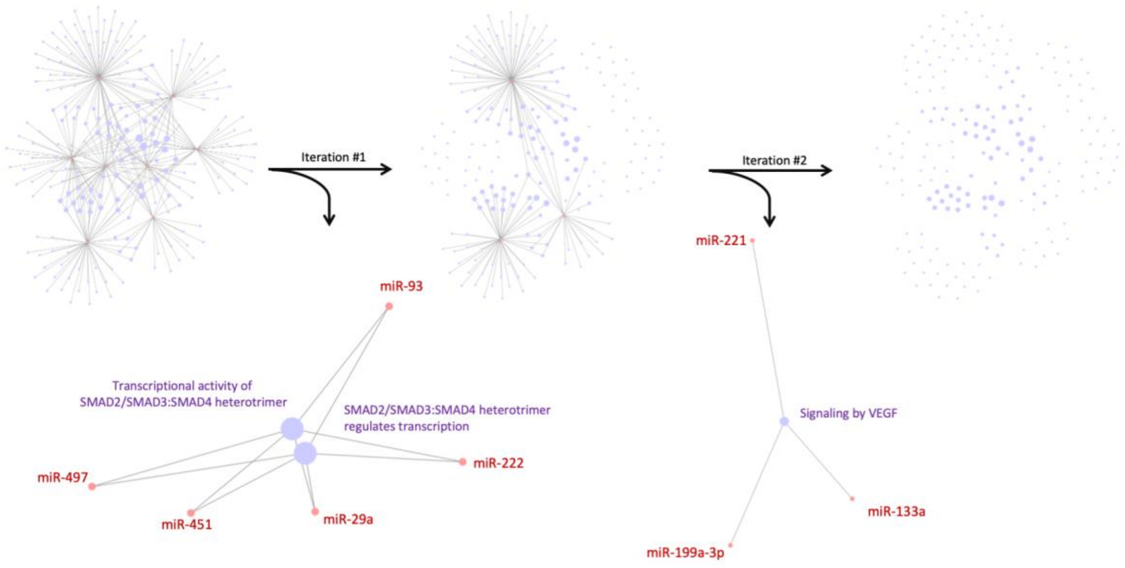

Figure 1. Two iterations of the Greedy search and pinpointed Reactome signaling pathways, regulated by studied human miRNAs. Red nodes indicate miRNAs, purple – Reactome pathways. Edges between miRNAs and Reactome pathways indicate that the key targets of these miRNAs are overrepresented in the corresponding pathways.

To test the statistical hypothesis whether the identified signaling pathways were pinpointed by chance, we estimated the probability of obtaining similar results with eight random miRNAs using the Monte Carlo method (100 iterations). The expected number of signaling pathways regulated by five miRNAs is estimated approximately three times less than that was actually found, and the number of signaling pathways regulated by three miRNAs is approximately two times less (p-value = 9.6×10^{-9}).
