# Supplementary material for: MiRNA-Regulated Pathways for Hypertrophic Cardiomyopathy: Network-Based Approach to Insight into Pathogenesis
Source: Genes (Basel). 2021 Dec 18;12(12):2016. doi: 10.3390/genes12122016 (PMC8701189; doi:10.3390/genes12122016)
Supplement: Supplementary file 1 [file genes-12-02016-s001.zip › Suppl_7.pdf]

Supplementary File 7:

Analysis of miRNAs interactions with key targets included in the second module

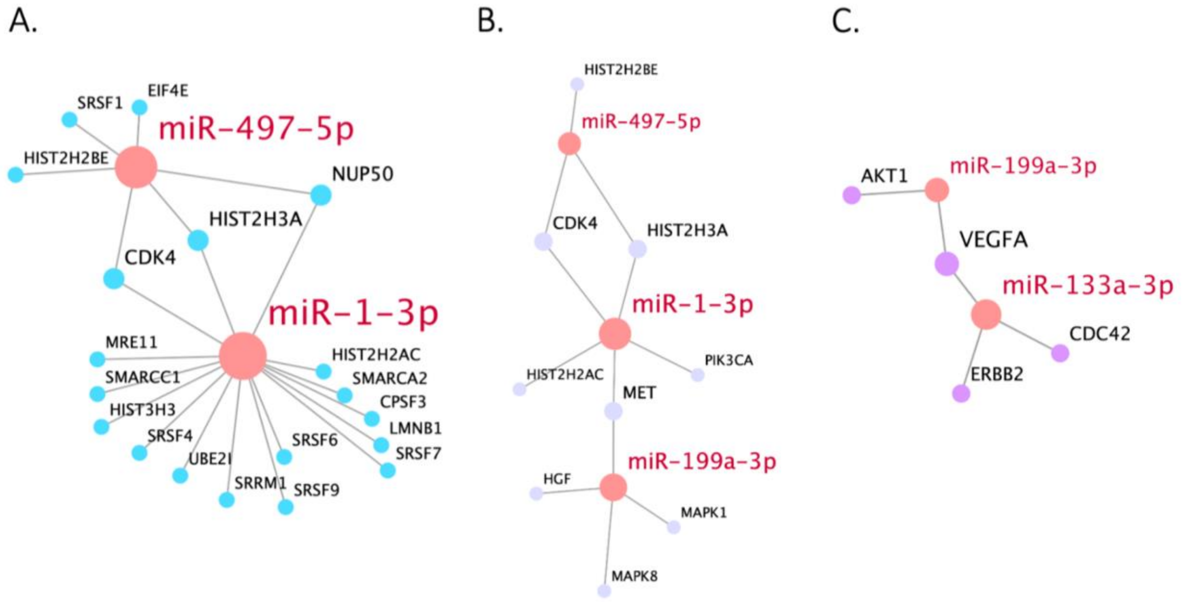

Figure 1S. Bigraphs of human miRNAs and their key target genes, overrepresented in the pathway cluster 1-4 (A), “bottleneck” pathways (B), and cluster 5-10 (C) from the second module.

As can be seen from Fig. 1S (A), hsa-miR-497-5p and hsa-miR-1-3p together regulate the activity of *CDK4*, *HIST2H3A*, and *NUP50* in the module capturing pathways 1-4 (see Fig. 2D). Thirteen other genes are targeted only by hsa-miR-1-3p, and three more – by hsa-miR-497-5p. In the “bottleneck” pathways of oxidative stress induced senescence and activation of PI3K/AKT signaling (Fig. 1S (B)), hsa-miR-1-3p interacts with the most number of genes. It has common targets with hsa-miR-497-5p (*CDK4* and *HIST2H3A* genes) and hsa-miR-199a-3p (*MET* gene). Beside these genes hsa-miR-1-3p targets *HIST2H2AC* and *PIK3CA*, hsa-miR-497-5p – *HIST2H2BE* and hsa-miR-199a-3p – *HGF*, *MAPK8*, and *MAPK1*. Fig. 3D shows that *VEGFA* is the common target for both hsa-miR-133a-3p and hsa-miR-199-3p, regulating pathways 5-10 (see Fig. 1S (C)). In addition to this gene hsa-miR-133a regulates the expression of *CDC42* and *ERBB2*, and hsa-miR-199a-3p – of *AKT1*.

Although the probability of identification of this module did not pass the significance level of 0.05, we should briefly discuss pathways composing it. While pathways 1-4, associated

with common cellular processes, seem to be non-specific, clusters of pathways 5-10, involved in HIF-dependent cellular response to hypoxia and VEGF signaling, may play a prominent role in HCM. “Signaling by VEGF" is another signaling pathway, regulated by miRNAs dysfunctional in hypertrophic myocardium. *VEGFA* expression of the central gene of the cluster, *VEGFA*, is well known to be activated under hypoxia conditions in order to promote vascularization of the affected tissue [1]. In mice with HCM induced by chronic pressure overload, increased VEGF level was shown to promote angiogenesis and regression of hypertrophy [2]. In plasma of patients with HCM, VEGF level was significantly increased; it also correlated with structural and functional parameters of hypertrophic myocardium [3]. Polymorphic variants of the *VEGFA* gene affecting its expression level are associated with the severity of HCM [4,5]. Thus, increased expression changes in of hsa-miR-133a, hsa-miR-221 and hsa-miR-199a-3p in hypertrophic myocardium may affect hypoxia induced down-regulate VEGF expression and signaling and therefore contribute to inhibit angiogenesis and promote HCM progression. Regulated by hsa-miR-199a-3p and hsa-miR-497-5p “bottleneck” pathway of oxidative stress induced senescence, closely linked to hypoxia, may also promote HCM and the subsequent heart failure [1].

1. Shibuya, M. Vascular Endothelial Growth Factor (VEGF) and Its Receptor (VEGFR) Signaling in Angiogenesis: A Crucial Target for Anti- and Pro-Angiogenic Therapies. *Genes & Cancer* **2011**, *2*, 1097–1105, doi:10.1177/1947601911423031.
2. Jiang, Y.; Reynolds, C.; Xiao, C.; Feng, W.; Zhou, Z.; Rodriguez, W.; Tyagi, S.C.; Eaton, J.W.; Saari, J.T.; Kang, Y.J. Dietary Copper Supplementation Reverses Hypertrophic Cardiomyopathy Induced by Chronic Pressure Overload in Mice. *Journal of Experimental Medicine* **2007**, *204*, 657–666, doi:10.1084/jem.20061943.
3. Pudil, R.; Vasatova, M.; Fucikova, A.; Rehulkova, H.; Rehulka, P.; Palicka, V.; Stulik, J. Vascular Endothelial Growth Factor Is Associated with the Morphologic and Functional Parameters in Patients with Hypertrophic Cardiomyopathy. *BioMed Research International* **2015**, *2015*, e762950, doi:10.1155/2015/762950.
4. Alkon, J.; Friedberg, M.K.; Manlhiot, C.; Manickaraj, A.K.; Kinnear, C.; McCrindle, B.W.; Benson, L.N.; Addonizio, L.J.; Colan, S.D.; Mital, S. Genetic Variations in Hypoxia Response Genes Influence Hypertrophic Cardiomyopathy Phenotype. *Pediatr Res* **2012**, *72*, 583–592, doi:10.1038/pr.2012.126.
5. Pieles, G.E.; Alkon, J.; Manlhiot, C.; Fan, C.-P.S.; Kinnear, C.; Benson, L.N.; Mital, S.; Friedberg, M.K. Association between Genetic Variants in the HIF1A-VEGF Pathway and Left Ventricular Regional Myocardial Deformation in Patients with Hypertrophic Cardiomyopathy. *Pediatr Res* **2021**, *89*, 628–635, doi:10.1038/s41390-020-0929-z.
6. Breitkreuz, M.; Hamdani, N. A Change of Heart: Oxidative Stress in Governing Muscle Function? *Biophys Rev* **2015**, *7*, 321–341, doi:10.1007/s12551-015-0175-5.
