# Supplementary material for: MiRNA-Regulated Pathways for Hypertrophic Cardiomyopathy: Network-Based Approach to Insight into Pathogenesis
Source: Genes (Basel). 2021 Dec 18;12(12):2016. doi: 10.3390/genes12122016 (PMC8701189; doi:10.3390/genes12122016)
Supplement: Supplementary file 1 [file genes-12-02016-s001.zip › Suppl_Tab_1.pdf]

Supplementary Table S1. MiRNAs, selected for the network analysis.

miRNA	Direction of change in expression level in hypertrophy	Biological material	Species (compared groups)*	Reference
miR-1-3p	downregulated	left ventricle tissue	human (HCM vs HC)	[1]
	downregulated	left ventricle tissue	human (HCM vs HC)	[2]
miR-19b-3p	downregulated	myocardium	human (aortic stenosis associated hypertrophy vs HC)	[3]
	downregulated	myocardium	human (HCM vs HC)	[4]
miR-21-5p	upregulated	left ventricle tissue	human (HCM vs HC)	[5]
	upregulated	ventriculi	mice (DBL with TnI-203/MHC-403 mutations vs non transgenic)	[6]
hsa-miR-29a-3p	downregulated	left ventricle tissue	mice (HCM vs HC)	[7]
	downregulated	myocardium	mice (transverse aortic constriction, TAC vs ctrl);<br>human (aortic valve stenosis vs HC)	[8]
hsa-miR-93-5p	downregulated	*in vivo*: heart tissue;<br>*in vitro*: cardiomyocytes	mice (*in vivo:* HCM vs HC; *in vitro:* cardiomyocytes treated with Isoproterenol and Aldosterone vs untreated)	[9]
	downregulated	cardiomyocytes	rats (*in vitro:* AngII-treated vs untreated cardiomyocytes)	[10]
hsa-miR-133a-3p	downregulated	left ventricle and atria tissues	human (HCM vs HC);<br>mice (aortic constriction induced hypertrophy vs control; Akt-transgenic vs control);<br>rats (exercised vs control)	[11]
	downregulated	cardiomyocytes	AngII-treated vs untreated cardiomyocytes	[12]
miR-155-5p	downregulated	myocardium	human (HCM vs HC)	[4]
	upregulated	left ventricle tissue	human (HCM vs HC)	[1]
hsa-miR-199a-3p	upregulated	myocardium	mice (AngII-treated vs untreated)	[13]
	upregulated	left ventricle tissue	human (HCM vs HC)	[2]

hsa-miR-221-3p	upregulated	myocardium	human (HCM vs HC)	[4]
	upregulated	left ventricle tissue	human (HCM vs HC), mice (TAC vs ctrl)	[14]
hsa-miR-222-3p	upregulated	ventricular cardiomyocytes	rats (physiological hypertrophy vs HC)	[15]
	upregulated	myocardium	human (HCM vs HC)	[4]
hsa-miR-451a	downregulated	left ventricle tissue	human (HCM vs HC)	[5]
	downregulated	myocardium	mice (TAC vs HC)	[16]
hsa-miR-497-5p	downregulated	*in vivo*: myocardium; *in vitro*: cardiomyocytes	mice (*in vivo*: aortic constriction induced hypertrophy vs control; *in vitro*: *in vitro:* AngII-treated vs untreated cardiomyocytes)	[17]
	downregulated	*in vivo*: mycardium; *in vitro*: cardiomyocytes	rats (*in vivo*: aortic constriction induced hypertrophy vs control; *in vitro*: *in vitro:* AngII-treated vs untreated cardiomyocytes)	[18]

* According to miRBase database miRNA sequences in studied animal models were identical to those in humans, except for miR-497-5p: in mice and rats it has extra nucleotide on the 3' end of RNA molecule.

1. Li, M.; Chen, X.; Chen, L.; Chen, K.; Zhou, J.; Song, J. MiR-1-3p That Correlates with Left Ventricular Function of HCM Can Serve as a Potential Target and Differentiate HCM from DCM. *J Transl Med* **2018**, *16*, 161, doi:10.1186/s12967-018-1534-3.
2. Palacín, M.; Reguero, J.R.; Martín, M.; Díaz Molina, B.; Morís, C.; Alvarez, V.; Coto, E. Profile of MicroRNAs Differentially Produced in Hearts from Patients with Hypertrophic Cardiomyopathy and Sarcomeric Mutations. *Clin Chem* **2011**, *57*, 1614–1616, doi:10.1373/clinchem.2011.168005.
3. Beaumont, J.; López, B.; Ravassa, S.; Hermida, N.; San José, G.; Gallego, I.; Valencia, F.; Gómez-Doblas, J.J.; de Teresa, E.; Díez, J.; et al. MicroRNA-19b Is a Potential Biomarker of Increased Myocardial Collagen Cross-Linking in Patients with Aortic Stenosis and Heart Failure. *Sci Rep* **2017**, *7*, 40696, doi:10.1038/srep40696.
4. Huang, D.; Chen, Z.; Wang, J.; Chen, Y.; Liu, D.; Lin, K. MicroRNA-221 Is a Potential Biomarker of Myocardial Hypertrophy and Fibrosis in Hypertrophic Obstructive Cardiomyopathy. *Bioscience Reports* **2020**, *40*, doi:10.1042/BSR20191234.
5. Song, L.; Su, M.; Wang, S.; Zou, Y.; Wang, X.; Wang, Y.; Cui, H.; Zhao, P.; Hui, R.; Wang, J. MiR-451 Is Decreased in Hypertrophic Cardiomyopathy and Regulates Autophagy by Targeting TSC1. *Journal of Cellular and Molecular Medicine* **2014**, *18*, 2266–2274, doi:10.1111/jcmm.12380.
6. Bagnall, R.D.; Tsoutsman, T.; Shephard, R.E.; Ritchie, W.; Semsarian, C. Global MicroRNA Profiling of the Mouse Ventricles during Development of Severe Hypertrophic Cardiomyopathy and Heart Failure. *PLoS One* **2012**, *7*, e44744, doi:10.1371/journal.pone.0044744.
7. Liu, Y.; Afzal, J.; Vakrou, S.; Greenland, G.V.; Talbot, C.C.; Hebl, V.B.; Guan, Y.; Karmali, R.; Tardiff, J.C.; Leinwand, L.A.; et al. Differences in MicroRNA-29 and Pro-Fibrotic Gene Expression in Mouse and Human Hypertrophic Cardiomyopathy. *Frontiers in Cardiovascular Medicine* **2019**, *6*, 170, doi:10.3389/fcvm.2019.00170.
8. Sassi, Y.; Avramopoulos, P.; Ramanujam, D.; Grüter, L.; Werfel, S.; Giosele, S.; Brunner, A.-D.; Esfandyari, D.; Papadopoulou, A.S.; De Strooper, B.; et al. Cardiac Myocyte MiR-29 Promotes Pathological Remodeling of the Heart by Activating Wnt Signaling. *Nat Commun* **2017**, *8*, 1614, doi:10.1038/s41467-017-01737-4.
9. Wo, Y.; Guo, J.; Li, P.; Yang, H.; Wo, J. Long Non-Coding RNA CHRF Facilitates Cardiac Hypertrophy through Regulating Akt3 via MiR-93. *Cardiovascular Pathology* **2018**, *35*, 29–36, doi:10.1016/j.carpath.2018.04.003.

10. Li, Y.; Wang, J.; Sun, L.; Zhu, S. LncRNA Myocardial Infarction-Associated Transcript (MIAT) Contributed to Cardiac Hypertrophy by Regulating TLR4 via MiR-93. *European Journal of Pharmacology* **2018**, *818*, 508–517, doi:10.1016/j.ejphar.2017.11.031.
11. Care, A.; Catalucci, D.; Felicetti, F.; Bonci, D.; Addario, A.; Gallo, P.; Bang, M.-L.; Segnalini, P.; Gu, Y.; Dalton, N.D. MicroRNA-133 Controls Cardiac Hypertrophy. *Nature medicine* **2007**, *13*, 613–618.
12. Zhu, Y.-F.; Wang, R.; Chen, W.; Cao, Y.-D.; Li, L.-P.; Chen, X. MiR-133a-3p Attenuates Cardiomyocyte Hypertrophy through Inhibiting Pyroptosis Activation by Targeting IKKε. *Acta Histochemica* **2021**, *123*, 151653, doi:10.1016/j.acthis.2020.151653.
13. Zeng, N.; Huang, Y.-Q.; Yan, Y.-M.; Hu, Z.-Q.; Zhang, Z.; Feng, J.-X.; Guo, J.-S.; Zhu, J.-N.; Fu, Y.-H.; Wang, X.-P.; et al. Diverging Targets Mediate the Pathological Roleof MiR-199a-5p and MiR-199a-3p by Promoting Cardiac Hypertrophy and Fibrosis. *Molecular Therapy - Nucleic Acids* **2021**, *26*, 1035–1050, doi:10.1016/j.omtn.2021.10.013.
14. Wang, C.; Wang, S.; Zhao, P.; Wang, X.; Wang, J.; Wang, Y.; Song, L.; Zou, Y.; Hui, R. MiR-221 Promotes Cardiac Hypertrophy in Vitro through the Modulation of P27 Expression. *Journal of Cellular Biochemistry* **2012**, *113*, 2040–2046, doi:10.1002/jcb.24075.
15. Liu, X.; Xiao, J.; Zhu, H.; Wei, X.; Platt, C.; Damilano, F.; Xiao, C.; Bezzerides, V.; Boström, P.; Che, L.; et al. MiR-222 Is Necessary for Exercise-Induced Cardiac Growth and Protects against Pathological Cardiac Remodeling. *Cell Metab* **2015**, *21*, 584–595, doi:10.1016/j.cmet.2015.02.014.
16. Feng, H.J.; Ouyang, W.; Liu, J.H.; Sun, Y.G.; Hu, R.; Huang, L.H.; Xian, J.L.; Jing, C.F.; Zhou, M.J. Global MicroRNA Profiles and Signaling Pathways in the Development of Cardiac Hypertrophy. *Braz J Med Biol Res* **2014**, *47*, 361–368, doi:10.1590/1414-431x20142937.
17. Xiao, Y.; Zhang, X.; Fan, S.; Cui, G.; Shen, Z. MicroRNA-497 Inhibits Cardiac Hypertrophy by Targeting Sirt4. *PLOS ONE* **2016**, *11*, e0168078, doi:10.1371/journal.pone.0168078.
18. Zhang, G.; Ni, X. Knockdown of TUG1 Rescues Cardiomyocyte Hypertrophy through Targeting the MiR-497/MEF2C Axis. *Open Life Sciences* **2021**, *16*, 242–251, doi:10.1515/biol-2021-0025.
